# Writing proficiency in English as L2 in Spanish children with dyslexia

**DOI:** 10.1007/s11881-023-00278-4

**Published:** 2023-01-27

**Authors:** Marta Álvarez-Cañizo, Olivia Afonso, Paz Suárez-Coalla

**Affiliations:** 1 Department of Psychology, University of Valladolid, Paseo de Belén, 1, 47011 Valladolid, Spain; 2 Centre for Psychological Research, Oxford Brookes University, Oxford, UK; 3 Department of Psychology, University of Oviedo, Oviedo, Spain

**Keywords:** Writing, EFL, Spanish, Children, Dyslexia

## Abstract

Different studies have demonstrated that people with dyslexia have difficulties in acquiring fluent reading and writing. These problems are also evident when they learn a second language. The aim of our study was to investigate if there is a linguistic transfer effect for writing in children with dyslexia when they face tasks in English (L2), as well as the possible influence of other linguistic skills (spelling, vocabulary and reading) in English (L2) and in Spanish (L1). Participants completed a series of tasks both in Spanish and English: a picture naming task, a word reading task, a word spelling task, and a written composition of which we analysed its quality through different variables provided by the Coh-metrix software. Our results revealed that children with dyslexia show similar or parallel performance in written composition in both languages, which could imply a language transfer effect from L1 and L2. Besides, basic language skills are related to the characteristics of written composition to a greater extent in English than in Spanish, suggesting the impact of these on the quality of written composition.

## Introduction

A large body of literature has shown that children with dyslexia have difficulties learning to read and write (Hebert et al., 2018; Hulme & Snowling, 2009; Kemp, 2009; Suárez-Coalla & Cuetos, 2015). Furthermore, in terms of writing processes, it has been reported that individuals with dyslexia have problems to produce high-quality texts (e.g. Hebert et al., 2018; O’Rourke et al., 2020; Sumner & Connelly, 2020), and according to the *Linguistic Transfer Hypothesis* (*Linguistic Coding Deficits Hypothesis*, Sparks et al., 1989, 2012; *Linguistic Interdependence Hypothesis*, Verhoeven, 1994, and *Central Processing Hypothesis*, Cossu et al., 1988; Geva & Siegel, 2000; Stanovich, 1984), similar difficulties are to be expected when writing in English as a foreign or second language (L2). However, considering the complexity of the writing process, several skills and linguistic factors could potentially affect text quality, and they might not necessarily have the same influence when writing in the L1 (first language) and in the L2. In this context, the aim of this study is twofold. First, we try to investigate the similarities and differences between Spanish L1 and English L2 text compositions by Spanish children with dyslexia and test the *Linguistic Transfer Hypothesis* from writing skills in Spanish to writing skills in English; second, we explore the possible relationship between several language skills (namely, spelling, reading and vocabulary) and writing in both Spanish (L1) and English (L2). This is of great interest considering that English is the most widely spoken language in the world, making English writing truly useful for academic and professional purposes.

### Cognitive models of writing

Writing is a very complex activity, where many processes are involved. The writing model of Chenoweth and Hayes (2003) considers the existence of four levels: proposer, translator, transcriber and reviser. The proposer receives input from multiple processes of the model (long-term memory, social and physical influences) and generates ideas. The translator is responsible for turning these pre-linguistic ideas generated by the proposer into grammatical and ordered word strings. The transcriber converts the language generated by the translator into written language. The reviser assesses the output from the proposer and the translator, to ensure that the output matches the objectives of the writing goals. Text compositions are, therefore, a reflection of several processes and language skills. Consequently, difficulties in some of these levels will impact different aspects of the text, and maybe to a greater extent in L2 writing. On the other hand, depending on the level concerned, the difficulties to be observed may be more or less language-specific. If the problems are at the higher levels, involving more abstract processes (i.e. writing planning, knowledge activation or even attention), the problems should appear independently of the language. However, if the deficits imply lower or more linguistic levels of processing, such as spelling (transcriber) or grammar (translator), they should be more language-specific.

Although Chenoweth and Hayes’s influential model is aimed at describing the specific processes involved in writing, other authors have proposed models highlighting the close links between writing and reading. Despite of being different, these two processes are known to be bidirectionally related (Shanahan, 1984; Shanahan & Lomax, 1986), since they share common knowledge and skills (Fitzgerald & Shanahan, 2000). Fitzgerald and Shanahan (2000) established that readers and writers rely on four common knowledge bases: domain or content knowledge (specific knowledge of the topic they are reading or writing about); procedural knowledge (knowledge and skills needed for reading and writing, including relatively automatic processes, such as recalling information from memory or more intentional strategies such as predicting, summarizing or questioning); knowledge of specific features or components of written language (knowledge of features including phonemic, orthographic, morphological, lexical, syntactic and discourse features); and metalanguage (knowledge about functions and purposes of reading and writing). Hence, knowledge and skills learned in one modality may be applicable to the other because they underlie both the reading and writing process. However, the intricate relationship between reading, spelling and semantic is necessary to be considered in the writing context, to determine the possible influence of reading abilities on written compositions.

Numerous studies have shown that spelling and reading develop in a mutually dependent way (Caravolas et al., 2001; Conrad, 2008; Ehri, 2000; Ellis & Cataldo, 1990; Ritchey, 2008; Leppänen et al., 2006), and strong correlations have been found between word reading and spelling (Ehri, 2000). Spelling and reading are reciprocal tasks, involving the same cognitive skills. However, reading has also important connexions with other aspects of the writing activity, especially in the revision process. Thus, there are several levels of processing that might account for an influence of reading skills (and reading difficulties) on the quality of writing.

### Writing in dyslexia

In relation to dyslexia, it has been reported that the text compositions produced by people with dyslexia differ significantly from those produced by typically developing individuals (Afonso et al., 2022). Besides needing more time to produce texts (British Dyslexia Association, 2011; Rittle-Johnson & Siegler, 1999), several studies confirm that the written compositions of people with dyslexia include more spelling errors (Hebert et al., 2018; Morken & Helland, 2013), shorter sentences, less lexical diversity and less quality of ideas, than those by their peers (Afonso et al., 2022; Sumner et al., 2014; Wengelin, 2007). Particularly, Spanish children with dyslexia (4th to 6th grade) have been found to make a great number of errors when writing; in addition, they showed deficits in punctuation, syntactic and grammatical structure, and they write shorter sentences, with less lexical diversity (Afonso et al., 2022).

As mentioned above, it has been repeatedly pointed out that text quality depends, to a considerable extent, on spelling, and then, low-quality texts will be a consequence of spelling processes during text production. By having to concentrate on spelling retrieval (the orthographic representation of words), which takes time and cognitive resources away from the goal of writing, individuals with dyslexia would produce short and low-quality texts (Connelly, et al., 2006; Gregg et al., 2007; Sumner et al., 2014). In line with these ideas, children and adults with dyslexia exhibiting spelling difficulties show an impaired ability to encode, retain and access phonological information, which would impact on writing (Altemeier et al., 2008; Chan et al., 2006; Hebert et al., 2018; Uno et al., 2009). Additionally, children with dyslexia seem to need to rely more than children without dyslexia on reading processes when writing, so there is a relationship between reading time during writing and the quality of the text (Beers, Quinlan & Hardbaugh, 2010).

Moreover, according to the *triangular framework* (Seidenberg & McClelland, 1989) and the *lexical-quality hypothesis* (Perfetti & Hart, 2002), previous research has emphasized the benefits of high-quality semantic representations in reading (Nation & Snowling, 2004; Torppa et al., 2010) and spelling (Ouellette, 2010; Ouellette & Fraser, 2009). Therefore, vocabulary proficiency is also likely to have an impact on text quality, as a high level of vocabulary translates into greater lexical richness. But on top of that, vocabulary is clearly related to the amount of reading experience (Wise et al., 2007), and for people with dyslexia, as reading experience is often limited, the vocabulary level might be low, which would negatively affect written production (Olinghouse & Leaird, 2009).

Bearing in mind the difficulties exhibited by children with dyslexia, as well as the role of reading, spelling and vocabulary in written production, reading difficulties might not necessarily affect writing in the same way (i.e. through the same processes) when writing in the L1 than when writing in the L2. Thus, we aim to investigate in this study what happens when Spanish children with dyslexia write in English.

### Cross-linguistic transfer hypothesis and writing in L2

Text production in English could be a very demanding task for Spanish children, considering linguistic differences between English and Spanish, especially in relation to the characteristics of the orthographic system (Erickson & Sachse, 2010; Pagliuca et al., 2008). This challenge would be even greater for children with dyslexia, given their difficulties in L1, an aspect that has not been studied.

The *Linguistic Coding Deficits Hypothesis* (LCDH) (Sparks et al., 1989, 2012) and the *Linguistic Interdependence Hypothesis* (Verhoeven, 1994) predict that L2 learning is built on L1 skills, so writers who produce low-quality texts in L1 will also produce similar texts in L2. Furthermore, the characteristics of these texts will be related to the level of spelling, vocabulary and reading. Following the *Central Processing Hypothesis* (Cossu et al., 1988; Geva & Siegel, 2000; Stanovich, 1984), there are some underlying cognitive processes (e.g. verbal short-term memory and serial naming) and linguistic components (e.g. phonological skills), which role is fundamental in the emergence of reading skills in L1 and L2. A person with difficulties affecting linguistic skills (such as those affecting individuals with dyslexia) will also experience difficulties in acquiring reading skills, regardless of whether it is his L1 or L2. Besides, there will be differences between L1 and L2 performance if we consider the higher exposure to L1 versus L2. Since the exposure in L2 only occurs at school, while L1 occurs in different contexts. In addition, it is common to find that children with dyslexia have curricular adjustments in English class at school (Ministerio de Educación, Cultura y Deporte, 2012), and do not usually go to extracurricular English classes.

There are few studies focusing on the relationship between written composition ability in L1 and L2; but a study conducted with typically developing high school students, comparing written compositions in Dutch (L1) and English (L2), showed differences between L1 and L2 in terms of text quality and complexity, despite being two languages with great similarities (Tillema et al., 2013).

As far as people with dyslexia are concerned, there are no studies comparing written compositions in L1 and L2; however, there are some studies about other language skills in L1 and L2, which could be related to writing. Specifically, poor reading skills in English as L2 have been found in studies with participants with different L1: Polish (Jurek, 2004; Nijakowska, 2010), Norwegian (Helland & Kaasa, 2005; Helland & Morken, 2016), Swedish (Lindgren & Laine, 2012) and Spanish (Suárez-Coalla et al., 2020). Similar results were described for spelling in Italian (Bonifacci and Tobia, 2017; Palladino et al., 2016), Polish (Lockiewicz & Jaskulska, 2016), Chinese (Chung et al., 2020) and Spanish (Morente, 2020). However, we have not found any studies about the quality of compositions in English as L2.

### Current study

Given the importance of writing skills in English L2 and the lack of studies about Spanish children with dyslexia, the aim of our study was to investigate whether there is a linguistic transfer effect for writing in children with dyslexia when they face English writing. In addition, it is also intended to know the influence of certain skills related to writing, such as reading, spelling and vocabulary level in both Spanish as L1 and English as L2 written composition. We should not forget the relationship between these skills and their importance in written composition.

To this aim, Spanish children with dyslexia were asked to perform the same tasks in both Spanish and English languages: word reading, spelling-to-dictation, picture naming and a written composition about their hobbies or their family. These tasks were chosen for several reasons. In the first place, a reading task was conducted due to its clear relationship with spelling and writing, and because it is considered one of the main difficulties of children with dyslexia. As for spelling, we have already mentioned that children with dyslexia make numerous phonological and orthographic errors when writing, and the reported effect on text quality. Finally, vocabulary was chosen as another of the tasks, since lexical knowledge allows for more effective reading, but it can also influence writing. In relation to our first objective, we expect to find positive correlations between Spanish and English text characteristics, with better performance in Spanish due to the greater level of proficiency in the Spanish language. In relation to our second objective, which aims to find out the relationship between basic language skills and written composition, we expect some differences between languages due to differences in proficiency and the limited exposure of children with dyslexia to the English language. To this aim, we compared the results obtained in the reading words and writing words and picture naming tasks with the quality variables of the written compositions.

## Method

### Participants

Twenty-two children with dyslexia participated in this study (9 girls and 13 boys; (*M*_*age*_ = 11.69, *SD* = 2.14). They were all diagnosed as having dyslexia by skilled school counsellors or speech therapists. All subjects were within normal range on IQ (all participants with a score above 87 in WISC-IV or WISC-V) and participated in regular classes. In addition, all received or had received specific intervention for their reading difficulties. All participants came from a similar medium socio-economic background. All children were native speakers of Spanish, and none of them had lived in an English-speaking country. None of the participants spoke languages other than Spanish at home. In Spain, English learning starts at very early age (3 years old) in Spain by law (LOE 2/2006, 3rd May; LOMCE 3/2020, 29th December): they were exposed to formal teaching of oral English language from the 1st grade of preschool education (3 years old), and in primary school (from 6 years old), they were introduced to written English.

Out of the 22 total participants, 11 attended a Spanish–English bilingual school (*M*_*age*_ = 11.94, *SD* = 1.83) and 11 attended a Spanish monolingual school (*M*_*age*_ = 11.53, *SD* = 2.36). These bilingual schools have Spanish teachers and children attend 4 h of English lessons per week and follow a Content and Language Integrated Learning methodology (CLIL; Martínez-Agudo, 2019). Besides, 10 participants (four attending a bilingual school and six attending a monolingual school) received extra English classes (*M*_*age*_ = 12.21, *SD* = 2.45) and the other 12 did not have extra English classes (*M*_*age*_ = 11.25, *SD* = 1.83). Extra English classes are usually given in smaller groups than in schools, in some cases even individually. They can be reinforcement classes on what is seen at school, or they can introduce new content and themes.

Although one might think that there could be differences marked by the type of school attended, in bilingual schools, children with dyslexia receive all content in L1 or with adaptations, so there are no significant differences in L2 exposure.

Before starting the experimental tasks, the families of the participants received information about the purpose of the study, the type of task and its duration. A written informed consent was received from them, authorizing the students to take part in the experiment.

### Materials

Several tasks were performed in both Spanish and English: oral picture naming, word reading, spelling-to-dictation and writing composition.

#### Picture naming task

The vocabulary level was evaluated using two scaled tests of picture naming: the vocabulary task of Boston test (Goodglass et al., 1996) in Spanish, and the MINT test (Gollan et al., 2012) in English. In both, a series of black and white pictures were presented one by one, and children were asked to name them. Participants were given 10 s to produce a spontaneous response that could be increased up to 20 s if the participant ensured to know the word. If they did not remember the word, but recognized the object, a phonetic clue was offered (e.g. “the word starts with the sound…”).

#### Word reading aloud task

We selected 20 words with 5–6 letters in each language. Spanish words were selected according to their frequency of occurrence in children’s books (Martínez & García, 2008). For the English tasks, words were selected attending to the frequency of occurrence in English textbooks for Spanish-speaking children in a database (Martínez-García, Cuetos, Pérez-Litago, & Suárez-Coalla, in preparation). For each language, 10 words with high frequency and 10 words with low frequency were selected. We matched frequency across languages, so there are no significant differences between them. Mean word frequency of the words selected for this task in each language and *t* values comparing languages are given in Table 1.

**Table 1:** Frequency for reading aloud and spelling tasks in Spanish and English

	Frequency	Spanish task, *M (SD)*	English task, *M (SD)*	*p* values comparing languages
Reading aloud task	High	33.02 (4.8)	42.1 (18.5)	*p* = .174
	Low	8.72 (2.6)	6.7 (2.5)	*p* = .185
Spelling task	High	38.25 (4.1)	38.6 (13.6)	*p* = .940
	Low	6.99 (1.6)	8.2 (3.0)	*p* = .239

#### Word spelling-to-dictation task

A different set of 40 words (20 per language) were selected according to the same criteria applied for the reading aloud task (see Table 1).

#### Written composition

Participants were asked to produce a written composition in each language, based on a prompt (*Your hobbies or your family*). Participants had 2 min to plan their composition, after which they had to write it on a blank page with pencil or pen (they could choose the material with which they felt most comfortable and able to produce their best writing, so that the use of a pen or pencil would not be an impediment when writing the composition). All the children had a maximum time of 12 min to write each composition.

### Procedure

The procedure was approved by the Ethics Committee of Research of the Principality of Asturias and by the East Valladolid Ethics Committee of Research with Medication-Health Area and was carried out in accordance with The Code of Ethics of the World Medical Association (Declaration of Helsinki) for experiments involving humans. All the tasks were performed individually, in a silent room in a civic centre of the city. Participants carried out the different tasks in the same order (picture naming, reading aloud words, spelling words and writing composition, first in Spanish and then in English) in two sessions. Before performing each of the tasks, they were instructed to do it at their own pace, but without pausing. At the beginning of the first session, the participants and their families answered a brief questionnaire (see Appendix) about the type of school they attended, the number of English lessons that they had per week, the attendance to extra English classes and their level of exposure to the English language (e.g. reading or watching TV). Each session lasted about 45 min. To obtain information of the pauses and revisions made during the writing tasks, participants were video recorded with a Nikon D3200 camera, placed on a tripod on the table, so that the pen strokes made could always be seen.

### Analysis

The following measures were considered and analysed separately for each language.

1. *Picture naming*: accuracy, as the percentage of words correctly named
2. *Word reading task*: accuracy (number of words correctly read) and speed (total time to complete the task, expressed in seconds)
3. *Word spelling task*: accuracy, the number of words correctly spelled
4. *Writing compositions task:*
  4.1 *Speed: *number of words written in a minute, considering the time taken by each child to actually write the composition, rather than the maximum total time allowed for this task*.*
  4.2 *Errors*: percentage of spelling and grammar errors from the total number of written words.
  4.3 *Pauses and revisions*: we considered a pause when the child stopped writing, lifting the pencil or not; revisions included crossing out text, adding text, correcting spelling, correcting letter formation, correcting punctuation and capitalization and correcting grammar. This classification was taken from Sumner and Connelly (2020). Measures of pauses and revisions considered the text length (number of revisions or pauses/total words).
  4.4 Several measures of text complexity, obtained with Coh-Metrix for compositions in English (Graesser et al., 2004) and Coh-Metrix-Esp for compositions in Spanish (Quispersaravia, Perez, Sobrevilla, & Alva-Manchego, 2016). When transcribing the texts, only spelling errors were corrected. Coh-Metrix has been used by other authors for the judgements of essay quality (see Crossley & McNamara, 2011, for a review). This software allows obtaining information about the quality of a written text through the assessment of different variables. The measures used in our study are specified below:
    a **Descriptive indices**: number of words, sentences and paragraphs, and sentence length (number of words per sentence).
    b **Word information**: incidence (per 1000 words) of adverbs, adjectives, nouns, pronouns and verbs.
    c **Connectives**: incidence of connectors (per 1000 words), including logic, adversatives, temporal, additive, positive and negative connectives.
    d **Referential cohesion**: the average number of sentences in the text that have noun, argument and stem overlap (from one sentence back to the previous sentence).
    e **Lexical diversity**: Guiraud’s index ($Type/\surd Token)$ which takes into account the length of the text. The advantages of this measure over other indices, such as Type-Token ratio (TTR), have been confirmed by many studies (Broeder, Extra & Von Hout, 1993; Van Hout & Vermeer, 1988; Vermeer, 2000).

## Results

The data obtained were analysed using SPSS.27 software. First, we compared languages’ performance from the basic linguistic tasks (i.e. picture naming, reading aloud words and spelling words), and besides, the measures obtained in the written composition task. Furthermore, we performed several Spearman correlations between languages’ performance and between the basic linguistic tasks and the composition’s measures.

### Language comparison: Spanish vs English

#### Basic linguistic tasks

A Wilcoxon signed rank test was performed to investigate language differences in the performance of basic linguistic tasks. There was a statistically significant difference between English and Spanish in reading accuracy, *Z* = 2.85, *p* = 0.004, *r* = 0.61, *Power (1-β)* = 0.39; spelling accuracy, *Z* = *2.85*, *p* = 0.004, *r* = 0.611, *Power (1-β)* = 0.39; and picture naming accuracy, *Z* = 4.108, *p* < 0.001, *r* = 0.876, *Power (1-β)* = 0.57 (see Table 2). As expected, children with dyslexia had a better performance in Spanish than in English.

**Table 2:** Mean and standard deviation of tasks by language

		Spanish, *M (SD)*	English, *M (SD)*
Basic linguistic tasks	Reading words accuracy	16.23 (2.89)	14.32 (4.15)
	Reading words speed	39.59 (26.52)	43 (39.25)
	Spelling words accuracy	14.5 (2.87)	7.41 (4.8)
	Picture naming accuracy	87.6 (31.08)	29.54 (13.72)
Compositions	Spelling errors	17.07 (14.07)	32.33 (26.48)
	Grammar errors	2.08 (2.47)	19.28 (15.97)
	Revisions	0.21 (0.26)	0.12 (0.88)
	Pauses	0.152 (0.07)	0.444 (0.23)
	Writing speed	15.07 (4.7)	9.22 (4.74)
	Number of words	68.18 (37.78)	46.64 (29.43)
	Number of sentences	3.59 (2.06)	4.45 (2.2)
	Number of paragraphs	2.32 (1.7)	2.73 (2.2)
	Sentence length	24.96 (22.36)	13.15 (9.71)
	Noun incidence	256.71 (67.45)	338.97 (130.2)
	Verb incidence	209.82 (42.75)	99.46 (73.05)
	Adjectives incidence	33.37 (25.25)	59.2 (35.44)
	Adverb incidence	46.58 (33.53)	21.57 (40.8)
	Pronoun incidence	53.9 (29.95)	162.42 (67.49)
	Connective incidence	79.48 (38)	76.82 (58.29)
	Noun overlap	0.18 (0.28)	0.15 (0.2)
	Argument overlap	0.5 (1.06)	0.43 (0.38)
	Stem overlap	0.18 (0.28)	0.18 (0.23)
	Lexical diversity	5.35 (0.97)	3.35 (0.89)

#### Written composition measures

A Wilcoxon signed rank test was performed to investigate language effect on the measures of written compositions. Significant differences were found between languages in spelling errors, *Z* = 3.215, *p* = 0.001, *r* = 0.686, *Power (1-β)* = 0.29; grammatical errors, *Z* = 4.107, *p* < 0.001, *r* = 0.876, *Power (1-β)* = 0.57; number of revisions, *Z* = 1.999, *p* = 0.046, *r* = 0.426, *Power (1-β)* = 0.44; number of pauses, *Z* = 4.107, *p* < 0.001, *r* = 0.876, *Power (1-β)* = 0.57; writing speed, *Z* = 4.074, *p* < 0.001, *r* = 0.869, *Power (1-β)* = 0.56; number of words, *Z* = 2.906, *p* = 0.004, *r* = 0.620, *Power (1-β)* = *0.37*; sentence length (mean number of words per sentence), *Z* = 3.020, *p* = 0.003, *r* = 0.644, *Power (1-β)* = *0.37*; noun incidence, *Z* = 2.808, *p* = 0.005, *r* = 0.599, *Power (1-β)* = *0.37*; verb incidence, *Z* = 3.912, *p* < 0.001, *r* = 0.834, *Power (1-β)* = *0.51*; adjective incidence, *Z* = 2.613, *p* = 0.009, *r* = 0.557, *Power (1-β)* = *0.39*; adverb incidence, *Z* = 2.311, *p* = 0.021, *r* = 0.493, *Power (1-β)* = *0.42*; pronouns incidence, *Z* = 3.945, *p* < 0.001, *r* = 0.841; and lexical diversity, *Z* = 4.042, *p* < 0.001, *r* = 0.862, *Power (1-β)* = *0.52*. Children with dyslexia had higher values in Spanish than in English for number of revisions, writing speed, sentence length, incidence of verbs, incidence of adverbs and lexical diversity. See Table 2 for a summary of these measures.

### Spearman correlations between languages

After finding, by means of the Shapiro–Wilk test, that more than half of the variables studied do not follow a normal distribution, we performed Spearman correlations between languages, to explore a possible cross-linguistic transference. In the basic linguistic tasks, we found significant positive correlations between languages in word reading accuracy, *r* = 0.771, *p* = 0.000; word reading speed, *r* = 0.683, *p* = 0.000; and word spelling accuracy, *r* = 0.617, *p* = 0.002; no significant correlation was found between picture naming accuracy in Spanish and English, *r* = 0.374, *p* = 0.086.

In addition, considering the text composition’s measures, significant positive correlations between languages were found: spelling errors, *r* = 0.527, *p* = 0.012; writing revisions, *r* = 0.422, *p* = 0.050; writing pauses, *r* = 0.484, *p* = 0.022; writing speed, *r* = 0.708, *p* < 0.001; number of words, *r* = 0.583, *p* = 0.004; number of sentences, *r* = 0.588, *p* = 0.004; and lexical diversity, *r* = 0.639, *p* = 0.001.

### Spearman correlations between basic linguistic tasks and composition measures by language

The relationship between basic linguistic tasks and composition measures was investigated using Spearman correlation coefficient, since data did not meet the requirements to use the parametric test.

In Spanish language, there were negative correlations between word reading accuracy and spelling errors, *r* =  − 0.481, *p* = 0.023, and writing speed, *r* =  − 0.623, *p* = 0.002, and a positive correlation with lexical diversity, *r* = 0.474, *p* = 0.026. Concerning word reading speed (i.e. the time that children took to read the list of words), it correlated negatively with number of words, *r* =  − 0.439, *p* = 0.041; number of sentences, *r* =  − 0.453, *p* = 0.041; noun overlap, *r* =  − 0.531, *p* = 0.011; stem overlap, *r* =  − . 531, *p* = 0.011; and lexical diversity, *r* =  − . 585, *p* = 0.004, in the writing task. Regarding spelling accuracy, we found significant positive correlations with noun overlap, *r* = 0.486, *p* = 0.022; stem overlap, *r* = 0.486, *p* = 0.022; and lexical diversity, *r* = 0.482, *p* = 0.023. Also, negative correlations were found between spelling accuracy and spelling errors in the writing task, *r* =  − 0.680, *p* = 0.000. Finally, picture naming accuracy correlated positively with pronoun incidence, *r* = 0.476, *p* = 0.025.

Concerning the English language tasks, we found positive correlations between word reading accuracy and number of words, *r* = 0.774, *p* < 0.001; sentence length, *r* = 0.623, *p* = 0.002; noun overlap, *r* = 0.546, *p* = 0.009; stem overlap, *r* = 0.504, *p* = 0.017; and lexical diversity, *r* = 0.581, *p* = 0.002; also, negative correlations were found between word reading accuracy and spelling errors, *r* =  − 0.629, *p* = 0.002; number of pauses, *r* =  − 0.437, *p* = 0.042; and noun incidence, *r* =  − 0.562, *p* = 0.006. Word reading speed correlated positively with spelling errors, *r* = 0.525, *p* = 0.012; number of pauses, *r* = 0.496, *p* = 0.019; and noun incidence, *r* = 0.580, *p* = 0.005, and negatively with number of words, *r* =  − 0.513, *p* = 0.015; noun overlap, *r* =  − 0.458, *p* = 0.032; stem overlap, *r* =  − 0.470, *p* = 0.027; and lexical diversity, *r* =  − 0.533, *p* = 0.011. Concerning spelling accuracy, we found positive correlations with number of words, *r* = 0.500, *p* = 0.018; argument overlap, *r* = 0.424, *p* = 0.049; and lexical diversity, *r* = 0.451, *p* = 0.035; negative correlations were found with spelling errors, *r* =  − 0.766, *p* = 0.000; pauses, *r* =  − 0.450, *p* = 0.035; and noun incidence, *r* =  − 0.449, *p* = 0.035. Finally, picture naming accuracy was found to correlate positively with number of words, *r* = 0.867, *p* < 0.001; sentence length, *r* = 0.473, *p* = 0.026; connector incidence, *r* = 0.482, *p* = 0.023; noun overlap, *r* = 0.579, *p* = 0.005; argument overlap, *r* = 0.586, *p* = 0.004; stem overlap, *r* = 0.535, *p* = 0.010; and lexical diversity, *r* = 0.768, *p* < 0.001, and negatively with spelling errors, *r* =  − 0.428, *p* = 0.047, and noun incidence, *r* =  − 0.427, *p* = 0.048.

## Discussion

The aim of our study was to investigate the similarities and differences between Spanish L1 and English L2 text compositions by Spanish children with dyslexia. Besides, we wanted to know the influence of certain basic linguistic skills (i.e. reading accuracy, spelling accuracy and vocabulary level) on the written compositions in Spanish L1 and English L2. To do this, Spanish children with dyslexia completed a series of tasks in both Spanish and English languages, including picture naming, word reading aloud and word spelling-to-dictation tasks as well as a short composition task writing about their hobbies or family.

Regarding the first objective, the results suggested that there are important similarities between languages when text composition is considered. Specifically, we found a positive correlation between languages in the number of spelling errors, writing revisions, writing pauses, writing speed, number of words, number of sentences and lexical diversity. It seems to indicate that children with dyslexia show similar or parallel performance in written composition in both languages, which could imply a language transfer effect from L1 and L2 (Cossu et al., 1988; Geva et al., 2000; Sparks et al., 1989, 2012; Stanovich, 1984; Verhoeven, 1994). Several authors showed that writing in children with dyslexia in their L1 is characterized by many spelling errors, a low writing speed due to the frequent pausing (Sumner et al., 2014) and poor lexical diversity (British Dyslexia Association, 2011; Coleman et al., 2009; Connelly et al.,; 2006; Crossley et al., 2009; Gregg et al., 2007; Rittle-Johnson & Siegler, 1999; Sumner et al., 2014). According to the linguistic transfer framework, similar features could be found in the L2. In this study, we found that Spanish children who are more productive, more accurate and faster when writing in their first language are also so when they write in English. Interestingly, lexical diversity also significantly correlated between languages. Thus, basic transcription skills seem to transfer to the L2 as well as the effects of these skills on word selection previously reported in the literature. In this context, our results are in line with the predictions of the *Linguistic Coding Deficit Hypothesis* (Sparks et al., 1989, 1991, 1993, 1995) and the *Linguistic Interdependence Hypothesis* (Verhoeven, 1994). The *Linguistic Coding Deficit Hypothesis* proposes that the difficulties in L2 can be explained by the difficulties they have in their native language. Thus, the difficulties that children with dyslexia have in Spanish, their native language, are transferred when they perform a task in a second language, such as English. On the other hand, the *Linguistic Interdependence Hypothesis* considers that L2 learning is built on L1 skills, so writers who produce low-quality texts in L1 will also produce similar texts in L2.

On the other hand, the language comparison analysis showed some differences between languages, with a better performance in most measures of Spanish compositions. Firstly, fewer mistakes, both in spelling and grammar, and pauses during the Spanish writing were found. However, analysis showed more revisions during the Spanish composition. This means that when they write in their L1, they read it more often than in L2; this is likely to be due to their greater experience in writing in L1. Spanish children are more used to writing in L1 and may have been instructed to do so throughout their school life. Alternatively, children may have more time to revise their composition when writing in Spanish than in English, as they write faster and with fewer pauses in their L1. As regards to quality of the composition, we found larger compositions in terms of total number of words and words per sentence in Spanish, in addition to higher lexical diversity. Furthermore, we found a higher incidence of nouns, adjectives and pronouns in English than in Spanish, but higher incidence of verbs and adverbs in Spanish. This might indicate that children’s compositions in English are more descriptive of concrete objects and simple situations, while compositions in Spanish would include more complex situations involving more actions and qualifiers of these actions. However, it is important to notice that the case of pronouns can be attributed to the grammatical characteristics of English, in which the verb always must be accompanied by a pronoun, which is not the case in Spanish. A more extensive knowledge of nouns than of verbs might be a consequence of their English learning method, sometimes based in semantic fields. Therefore, in general terms, children with dyslexia performed better when they wrote in Spanish (L1) than in English (L2). Furthermore, it seems clear that the characteristics of the compositions differed according to the language. Previous studies conducted with university students comparing writing in L1 and L2 (English) showed that when they wrote in L2, the compositions had a simpler structure, using an excessively basic syntax and vague and general nouns (Hinkel, 2003). Bearing this in mind, we can explain the lower use of verbs in English in our study as a consequence of the use of simple syntax, which also would explain the greater number of sentences (non-significant difference) observed in English, since a string of simpler, shorter sentences would be produced in English to explain the same ideas expressed in the more complex sentences produced in Spanish.

As regards the second objective, which addressed the relationship between basic linguistic tasks (reading, spelling and picture naming) and composition measures, we found interesting results. In Spanish language, reading accuracy was related with spelling errors and lexical diversity. Thus, children with better performance in reading committed fewer spelling errors, which is in accordance with previous studies about the strong relationship between reading and spelling (Caravolas et al., 2001; Conrad, 2008; Ehri, 2000; Ellis et al., 1990; Ritchey, 2008; Leppänen et al., 2006). In addition, children with better level in reading seem to exhibit more lexical diversity when writing. On the other hand, reading time correlated negatively with number of words and sentences, noun and stem overlap and lexical diversity. This association might be related to revision processes. Slow readers might not be able to review their text as effectively as faster readers, or it might take them more time to do so. Although some authors have proposed that this might be the case (Sumner & Connelly, 2020), more research is necessary to clarify the relationship between reading and editing processes during revision in writing.

Regarding spelling accuracy, we found that children with better spelling level committed fewer mistakes during text composition and showed more noun overlap, stem overlap and lexical diversity. Of course, a correlation does not imply causality, but it has often been reported that spelling has a huge impact on text quality. Individuals with dyslexia might produce lower quality texts (Connelly, et al., 2006; Gregg et al., 2007; Sumner et al., 2014) because spelling retrieval consumes excessive cognitive resources for these writers than for writers without dyslexia.

As for lexical diversity, it should be noted that vocabulary is known to be related to reading experience, and this is often limited in people with dyslexia, impacting negatively on written production (Olinghouse & Leaird, 2009). In addition, these findings could confirm that problems experienced by participants with dyslexia affect the lexical diversity. It has even proposed (Afonso et al., 2022) that this reduced lexical due to the high cognitive cost of transcription might be the ultimate cause of the low-quality scores given to compositions written by students with dyslexia.

Concerning the English language tasks, we found that both better reading accuracy and higher reading speed were related to fewer spelling errors, number of pauses and noun incidence and with a greater number of words, higher noun and stem incidence and lexical diversity. Reading accuracy was additionally associated to the production of longer sentences. Therefore, there is a relationship between reading and writing, but also with quality of written compositions, in terms of number of words and sentences and words per sentence, as was seen in Japanese high school students (Ito, 2011). We have also found that low speed for reading words is related to more errors in writing, as well as more pauses, fewer words and less lexical diversity, among others. As mentioned above, it seems that a lower reading speed is related to a lower quality in the writing of compositions. Although these effects may be revealing poorer reviewing processes in slow readers, it might be low reading experience hinders the development of fluent reading, which, as it has been already mentioned, might be related to poorer vocabulary, which is reflected in lower lexical diversity and problems to spell the words correctly.

As regards spelling accuracy, we found that better performance was related with a greater number of words, argument overlap and lexical diversity, as well as with fewer spelling errors in the composition, number of pauses and noun incidence. In the literature, we could see some studies that showed how spelling errors are a good predictor of the quality of L2 written compositions (Bestgen et al., 2011; Harrison et al., 2016). Finally, picture naming accuracy was found to correlate positively with number of words, sentence length, connector incidence, noun overlap, argument overlap, stem overlap and lexical diversity and negatively with spelling errors and noun incidence. In this case, we see that vocabulary plays a much more important role in English than in Spanish. Students used to claim that the biggest difficult in L2 is their lack of L2 vocabulary (e.g. Aliakbari, 2002). Nation (2001) considers vocabulary as more challenging in writing than in reading, as a productive use of words needs more linguistic knowledge, more practice and a stronger semantic association, in addition to a higher motivation to use.

As can be seen, basic language skills are related to the characteristics of written composition to a greater extent in English than in Spanish. It has already been seen that writing in L2 is more demanding than writing in L1 (Roca de Larios et al., 2011; Schoonen et al., 2003; Thorson, 2000; Van Weijen et al., 2008), as it requires to lean on more skills. Besides, in the case of children with dyslexia, whose English level would probably be reduced, this demand is even higher. These data suggest the extent to which reading, spelling and vocabulary skills can impact the quality of writing. It has been reported that spelling impacts writing quality, but the relationship between reading, spelling and semantic is clear in our study. The differences between languages on the effect of basic language tasks (with lower performance in English than in Spanish) suggest that these basic linguistic skills such as reading and transcription abilities affect the characteristics of written compositions to a larger extent when these skills are more limited.

### Limitations and future directions

Our study highlights the differences between writing in L1 and L2 in Spanish children with dyslexia. This is a first step that could allow us to learn more about the scope of the difficulties of these children, which might help to design more effective interventions in this population. Although it is not necessary for the purpose of our study, it could be interesting to have a group of children without dyslexia, to check if the same differences between languages exist. In the same way, the tasks that were carried out could have also included other aspects such as working memory or even a possible diagnosis of developmental coordination disorder, although none of the participants reported having motor difficulties which may also influence their writing. Finally, we can point out the reduced sample size tested as a limitation. Although our findings remain of interest, it is important to bear in mind that larger studies should confirm the trends reported here. In the same way, it would be pertinent to analyse in the future the influence of the English teaching methodology on writing in this language as L2, for example comparing monolingual schools and bilingual schools, including attendance at extra English classes.

Therefore, both the comparison with a control group (which can give us more information about the real difficulties that children with dyslexia have when dealing with a second language) and the expansion of initial tests that include working memory or motor coordination difficulties (which can significantly alter the writing) could be future directions of research to consider, due to the limited knowledge about writing in English as L2 in Spanish children with dyslexia.

## Data Availability

The data of our study can be sent on request.
